# Innovative Cold Plasma Pretreatment and Enzyme-Assisted Extraction of Genistein from Edamame and Storage Stability of Dried Extract Powder

**DOI:** 10.3390/foods14122118

**Published:** 2025-06-17

**Authors:** Shaher Bano, Sarana Rose Sommano, Noppol Leksawasdi, Siraphat Taesuwan, Pornchai Rachtanapun, Charin Techapun, Nutsuda Sumonsiri, Julaluk Khemacheewakul

**Affiliations:** 1 Division of Food Science and Technology, School of Agro-Industry, Faculty of Agro-Industry, Chiang Mai University, Chiang Mai 50100, Thailand; shaher_bano@cmu.ac.th (S.B.); siraphat.t@cmu.ac.th (S.T.); 2 Plant Bioactive Compound Laboratory (BAC), Department of Plant and Soil Sciences, Faculty of Agriculture, Chiang Mai University, Chiang Mai 50200, Thailand; sarana.s@cmu.ac.th; 3 Bioprocess Research Cluster, School of Agro-Industry, Faculty of Agro-Industry, Chiang Mai University, Chiang Mai 50100, Thailand; noppol.l@cmu.ac.th (N.L.); charin.t@cmu.ac.th (C.T.); 4 Center of Excellence in Agro Bio-Circular-Green Industry (Agro BCG), Faculty of Agro-Industry, Chiang Mai University, Chiang Mai 50100, Thailand; pornchai.r@cmu.ac.th; 5 School of Health and Life Sciences, Teesside University, Middlesbrough TS1 3BX, UK; n.sumonsiri@tees.ac.uk

**Keywords:** cold plasma, enzyme, extraction, isoflavone, green soybean

## Abstract

Green soybeans, or edamame (*Glycine max* L. Merril), serve as a superior source of phytochemicals and other nutritive substances and are commonly used as ingredients and additives in food products due to their polyphenols’ functional properties and antioxidant activity. Hence, it is very important to use a process to extract compounds with functional roles from plants as efficiently as possible. In this study, we sought to identify the optimal conditions for extracting genistein, belonging to the aglycone subgroup of isoflavones, from edamame using the cold plasma (CP) and enzyme method. Additionally, the impact of various drying techniques (spray-drying and freeze-drying) and storage conditions on the crude genistein extract powder was evaluated. The findings showed that the maximum values for the total phenolic content (TPC), total flavonoid content (TFC), and genistein (22.5 ± 0.23 mg of gallic acid equivalents (GAE)/100 g; 15.3 ± 0.13 mg of catechin equivalents (CAE)/100 g; and 12.6 ± 0.10 mg/100 g, respectively) were achieved under optimal pretreatment conditions using a CP gas flow rate of 5 L/min for 30 min, followed by enzymatic treatment at a specific enzyme concentration of 2.0% (*v*/*v*) for 240 min of incubation. Moreover, a scanning electron microscopy (SEM) analysis demonstrated that the CP and enzyme treatment induced significant structural changes, as evidenced by the presence of deeper pores on the surface of the powder granules. Spray-drying demonstrated a superior efficacy compared to freeze-drying for encapsulating the crude isoflavone extract. This study’s results also demonstrated that storage at 4 °C significantly stabilized the TPC, TFC, and genistein content and the antioxidant activity while preserving the physical properties (solubility and color) of the crude extract powder for up to 45 days. In summary, cold plasma pretreatment and enzymatic treatments offer practical solutions by enhancing the efficiency of non-thermal extraction processes, thereby increasing the yield of bioactive compounds, maintaining quality, and diminishing reliance on traditional, harsh methods. The elevated genistein content in the crude extract powder indicates its prospective application as a functional ingredient in various food and nutraceutical contexts.

## 1. Introduction

Edamame, the immature green form of soybean (*Glycine max* L. Merrill), is widely regarded as a vegetable-type soybean due to its harvest at an early developmental stage. In recent years, its consumption has increased significantly in Asia and Europe, driven by the increasing awareness of its high nutritional value and potential health-promoting properties [1]. The nutrient composition of edamame is primarily specified by its chemical constituents, including carbohydrates, proteins, calcium, and phosphorus [2]. Phytochemicals, including saponins, isoflavones, and vitamins A, C and E, present in edamame, act as antioxidants, anti-inflammatory agents, and antimicrobials, which contribute to wound healing [3]. Edamame is also reported to be a rich source of isoflavones. Due to these superior nutritional components, edamame may confer health benefits, such as diminishing the potential impact of cancer, cardiovascular disease, and osteoporosis [4].

Isoflavones are polyphenolic compounds belonging to the flavonoid class. They are also considered a common category of phytoestrogens because of their similarities in structure to female estrogens. Isoflavones are commonly found in legumes such as soybeans, beans, chickpeas, and sunflower seeds. The major isoflavones found in soybeans are genistein, daidzein, and glycitein, present at concentrations ranging between 1.2 and 4.2 mg/g dry weight [5]. These compounds possess antioxidant properties and can benefit human health by reducing the risk of cancer, obesity, diabetes, cardiovascular disease, and osteoporosis and alleviating menopausal symptoms [6]. According to Kim [7], soybeans possess the ability to prevent and eliminate carcinoma, owing to their elevated genistein content. The process of extracting isoflavones from botanical sources is an essential phase in the development of effective and economical methods [8].

Isolating isoflavones from plant materials offers significant benefits and advantages, particularly in applications like pharmaceuticals, functional foods, and cosmetics. An optimal extraction method should meet several criteria, including simplicity, safety, reproducibility, cost-efficiency, and suitability for large-scale industrial implementation [9]. Critically, the chosen method must also maintain the integrity of the native isoflavone profile [8]. In this context, mild and non-thermal approaches, including cold plasma pretreatment and enzymatic-assisted extraction, have been recognized as potentially effective alternatives to facilitate the selective and gentle process of recovering compounds with biological activity.

Non-thermal extraction processes are methods that enhance mass transfer rates and increase the cell membrane permeability, allowing extractions to occur at or near room temperature. This process helps preserve thermosensitive compounds, leading to higher extraction yields and reduced degradation or contamination of the final [10]. Common non-thermal techniques employ ultrasound, high hydrostatic pressure, pulsed electric fields, microwaves, and cold plasma (CP) [11]. In contrast to ultrasound- or microwave-assisted extraction, CP functions at atmospheric pressure and near-room ambient temperatures and typically does not require additional solvents, making it a more environmentally friendly and sustainable approach [12]. Additionally, CP pretreatment enhances the permeability of plant cell structures, thereby improving the enzymes’ accessibility and increasing the efficiency of enzyme-assisted extraction when used in combination [13].

CP is a non-thermal technology capable of generating high-quality phytoextracts while minimizing degradation and side effects [14]. One of the main advantages of CP over traditional extraction methods is its ability to preserve the main plant components while exerting minimal influence over the product’s internal structure [15]. In the extraction industry, CP represents an environmentally sustainable method that generates no toxicity or potentially hazardous residues and does not require water or solvents—an advantage for the generation of excellent-quality extracts from plants [16]. Tripathy and Srivastav [17] reported that the pretreatment of *Centella asiatica* L. with CP, in the form of cold plasma-activated water (CPAW), significantly (*p* < 0.05) increased the antioxidant activity in comparison with the control. The CPAW pretreatment notably softened the cellular structure of the leaf surface, leading to cell shrinkage, increased intracellular space, and the formation of larger pores. Since proteins are present in plant cell walls and contribute to cellular structure and function, the CP pretreatment can alter their three-dimensional conformation by cleaving the bonds between peptides in the presence of nitrogenous species that are highly susceptible to the reaction. This structural disruption may account for the softened cell walls, thereby facilitating more efficient extraction of the compound [18].

Wang et al. [19] also observed enhanced antioxidant release due to the breakdown of peanut cell walls during CP pretreatment. Similarly, Rout and Srivastav [20] found that both the flavonoid and phenolic concentrations became elevated with extended treatment with plasma durations at voltages of 30 kV and 35 kV for 4 and 6 min, respectively. However, like all emerging technologies, further research is needed to optimize CP for the efficient extraction of natural antioxidant compounds at an industrial scale [16].

Enzyme-assisted extraction is an innovative and environmentally sustainable technique that is extensively employed as an environmentally sustainable approach for improving phytochemical yields from various plant matrices, primarily due to its use involving water as an extraction solvent instead of solvents containing organic compounds [21]. Various enzymes, including α-amylase, pectinase, and cellulase, are employed for hydrolyzing polysaccharides and cell walls in plant material. Additionally, factors including the concentration and composition of the enzyme, the ratio of sample to solvent, the hydrolysis duration, the particle size, and the amount of moisture play critical roles in the extraction process [22]. Enzyme-assisted extraction has been effectively used for recovering various bioactive compounds, including anthocyanins, non-anthocyanin flavonoids, phenolics, and other antioxidants [23]. For example, the CP-assisted enzyme method has been effectively developed for extracting anthocyanins in haskap berries (*Lonicera caerulea* L.), demonstrating advantages including shortened extraction duration, decreased solvent consumption, and significantly enhanced levels of active substances along with antioxidant activity [24].

However, the combination of CP and enzymatic methods, which has demonstrated potential for efficiency and environmental sustainability, has not yet been reported for genistein extraction. Therefore, in this study, we aimed to analyze the optimal extraction conditions using CP and enzymatic methods for the extraction of genistein from edamame. The exterior characteristics of both the original and treated powder samples were examined by using scanning electron microscopy (SEM). Additionally, the impact of various drying methods (spray-drying and freeze-drying) and storage conditions on the bioactive compounds and their antioxidant activities was evaluated.

## 2. Materials and Methods

### 2.1. Raw Materials

Lanna Agro Industry Co., Ltd. (LACO, Chiang Mai, Thailand) provided the frozen fresh edamame. Subsequent to thawing, the seeds were immersed in tap water at room temperature for 1 min. A hot air oven (Memmert UF 110, Schwabach, Germany) was then used to dehydrate the soaked seeds at 60 °C for 48 h to the point that the moisture content decreased below 10% and a constant weight was achieved [25]. Edamame flour was obtained by grinding the seeds into a fine powder using a grinder (Green Evolution PG2500, Bangkok, Thailand) fitted with a 40-mesh sieve. After that, the ground edamame seed powder was packaged into vacuum-sealed aluminum foil bags and kept at −20 °C for subsequent analysis. An enzyme complex consisting of cellulase, xylanase, and pectinase was acquired at Reach Biotechnology Co., Ltd., Bangkok, Thailand. Every chemical reagent used was analytical (Sigma-Aldrich, Milan, Italy)- or high-performance liquid chromatography (HPLC)-grade (Merck Life Science, Darmstadt, Germany).

### 2.2. Cold Plasma Pretreatment

The dielectric barrier discharge (DBD) cell (Mini-smart, PLASMART., Co., Ltd., Daegeon, Republic of Korea) at the Science and Technology Park, Chiang Mai University, was used for CP treatment under atmospheric pressure. Two parallel electrodes—one powered and the other ground—as well as an RF power supply running at 13.56 MHz comprised the structure of the DBD plasma generator. At a velocity of 30 cm/s, the grounded electrode oscillated, and powder samples were positioned on an aluminum tray above it. To guarantee a constant discharge gap, a distance of 1 mm was set between the powered electrode and the sample tray. With argon (Ar) gas flowing at a rate of 8 L/min, plasma was produced at 150 W. To examine the effect of plasma gases, Powder samples (5 g) were placed on an aluminum tray positioned above the grounded electrode, which oscillated at a velocity of 30 cm/s. and treatment times ranged from 20 to 30 min using nitrogen (N_2_) as the feed gas at flow rates of 5, 7, and 9 L/min. In order to analyze the total phenolic content (TPC), total flavonoid content (TFC), isoflavone levels, and antioxidant activity, samples were vacuum-sealed in aluminum bags and kept at −20 °C following each treatment. Untreated samples were used as controls [26].

### 2.3. Enzyme Hydrolysis

The optimal CP pretreatment condition, as determined from Section 2.2, based on the highest concentrations of phytochemicals (TPC, TFC, and genistein) extracted from edamame, was subsequently followed by enzymatic treatment using a pectinase–xylanase–cellulase (PXC) complex at a 2:1:1 ratio. Enzymes were introduced upon achieving the desired condition (CP pretreatment 5 L/min for 30 min). Enzyme concentrations of 1%, 1.5%, and 2% (*v*/*v*) were evaluated at hydrolysis durations of 120, 180, and 240 min [27]. A constant-temperature shaker was used to incubate 10 g of CP pretreated powder at 50 °C after it had been suspended in 100 mL of 1 M acetate buffer at pH 5 for enzymatic hydrolysis [28]. In order to inactivate enzymes, the mixture was immersed in boiling water at 80 °C for 10 min following hydrolysis. After 15 min of centrifuging (Nüve NF400R, Ankara City, Turkey) the mixture at 8595× *g*, the supernatant was gathered and kept at 4 °C for additional TPC, TFC, isoflavone, and antioxidant analysis [24].

### 2.4. Drying Conditions

The optimal combination of CP pretreatment and enzymatic-assisted extraction was selected for further drying processes using either spray-drying or freeze-drying. After extraction, maltodextrin (10–12 DE) was added as a coating agent. An Ultra-Turrax (IKA, Burladingen, Germany) was used to homogenize a mixture of 200 g of maltodextrin and 1000 mL of treated extract for 5 min at 11,000 rpm. Both freeze-drying and spray-drying procedures were later performed using the resultant solution.

For freeze-drying, the extract was spread out on an aluminum tray to a thickness of approximately 1 cm, and it was frozen for 24 h at −18 °C. A freeze-dryer (LABCONCO, Kansas, MO, USA) was used to lyophilize the material for 34 h until a consistent weight was reached. The condenser temperature was maintained at −40 °C, and the chamber pressure was maintained at 0.133 mbar [25].

For spray-drying, the following conditions were used to spray-dry 1000 mL of extract: inlet temperature of 130 °C, outlet temperature of 48 °C, feed rate of 2 mL/min, aspirator rate of 70%, pump rate of 10%, and a 0.7 mm dispersing nozzle and 0.3 mm cleaning needle. The process was conducted for 5 h using a BUCHI Mini Spray Dryer (B-290, Flawil, Switzerland) as described by Khonchaisri et al. [25].

Dehydrated powders were vacuum-sealed in aluminum foil pouches and refrigerated. A subsequent evaluation was conducted to determine the phytochemical properties, antioxidant activity, powder yield, moisture content, and water activity [29].

### 2.5. Morphology of Dried Edamame Extract

A scanning electron microscope (SEM) (JSM-IT300, JEOL Ltd., Peabody, MA, USA) operating at 15 kV was used to analyze the morphological characteristics of freeze-dried extracts that had been untreated, CP-treated, and CP–enzyme combination-treated. Aluminum stubs coated with gold were used to mount the samples. The 500 and 3,000× magnification were used to capture SEM images.

### 2.6. Storage Stability Test of Edamame Extract Powder

The edamame extract powder was vacuum-sealed in aluminum foil bags and stored at 4 °C and 25 °C, following the optimal drying method. Intervals of 0, 15, 30, and 45 days were used to measure isoflavone and antioxidant activity [29].

### 2.7. Phytochemical Analysis

#### 2.7.1. Determination of Total Phenolic Contents

Spectrophotometric analysis was used to determine TPC [30]. Briefly, 5 mL of Folin–Ciocalteu’s phenol reagent, 9 mL of water, and 1 mL of extract sample were combined. The mixture was thoroughly mixed with 4 mL of a 7% Na_2_CO_3_ solution after 5 min. A UV spectrophotometer (Cary 60 Bio, UV–Vis, Kuala Lumpur, Malaysia) was used to measure the absorbance at 750 nm after the mixture was left in the dark for 1 h at room temperature (25 ± 2 °C). The gallic acid solution was prepared to construct the calibration curve, from which the TPC was determined through extrapolation. The phenolic compounds were estimated in triplicate. Gallic acid equivalents per gram of dried sample (mg GAE/g) were used to express the TPC.

#### 2.7.2. Determination of Total Flavonoid Content

The colorimetric approach was used when determining TFC [31]. A volume of 0.3 mL of 5% NaNO_2_ and 4 mL of distilled water were combined with 1 mL of extract. Then, 0.3 mL of 10% AlCl_3_·H_2_O was added after 5 min. Following this, 2 mL of 1 M NaOH and 2.4 mL of distilled water were introduced 1 min later. After thoroughly mixing the solution, the absorbance at 510 nm was measured. The results are presented as milligrams of catechin equivalents per gram of dried sample (mg CAE/g), with the standard curves being established using catechin (5–300 mg/kg).

#### 2.7.3. Determination of Genistein

Methanol–water (80:20) was used as the mobile phase in HPLC with a C18 column and flow rate of 1.0 mL/min to analyze the samples for genistein. The column temperature was kept at 40 °C, and the injection volume was 10 µL. The wavelength of photodiode array detection was 260 nm. The concentrations of solvents A and B were 0.1% trifluoroacetic acid and 0.1% methanol in acetonitrile, respectively [32]. Genistein content was determined based on a standard calibration curve. Milligrams of genistein per gram of extract was the unit of measurement used for the extraction study. To take into consideration changes in moisture content, the results of the drying study were reported as milligrams of genistein per gram of dry matter (DM).

### 2.8. Antioxidant Analysis

#### 2.8.1. Ferric-Reducing Antioxidant Power (FRAP) Assay

The method described by Tyug et al. [33] was used to determine the FRAP assay. Initially, 2.5 mL of a 10 mM TPTZ solution in 40 mM HCl, 2.5 mL of a 20 mM FeCl_3_ solution, and 25 mL of a 0.3 M acetate buffer (pH 3.6) were combined to generate a fresh FRAP reagent. The sample solution, 150 µL of water, and 1.5 mL of the FRAP reagent were subsequently combined and incubated in the dark at room temperature for 30 min. A spectrophotometer (Cary 60 Bio, UV–Vis, Kuala Lumpur, Malaysia) was used to measure the absorbance at 593 nm. A standard curve was plotted using ferrous sulfate (FeSO_4_.7H_2_O) at concentrations ranging from 10 to 100 µg/mL. A value of mg Fe (II) per g dry weight was used to express the results.

#### 2.8.2. Trolox Equivalent Antioxidant Capacity (TEAC) Assay

To prepare ABTS·+ for the TEAC assay, 7.4 mM ABTS (2,2′-azino-bis (3-ethylbenzothiazoline-6-sulfonic acid)) and potassium persulfate (K_2_S_2_O_8_, 2.60 mM) solutions were mixed, and the mixture was then incubated in the dark for 14 h. The TEAC assay was performed using the diluted mixture. After mixing 2.85 mL of ABTS⁺ solution with 150 µL of extract, the mixture was incubated for 2 h at 25 °C. The TEAC was calculated as mg Trolox per g dry weight by measuring the absorbance at 734 nm (Cary 60 Bio, UV–Vis, Kuala Lumpur, Malaysia) against a blank solution [34].

### 2.9. Powder Yield

Following the freeze-drying and spray-drying processes, the weight of the crude genistein extract powder was recorded. Equation (1) was employed to determine the powder yield, which is the ratio of the mass of dry powder collected after drying to the initial mass of solids in the feed solution for drying.

(1) $$\mathrm{Powder}\;\mathrm{yield}\;(\%)=\frac{Recovered\;solid\;mass}{Initial\;solid\;mass\;in\;feed}\times 100$$

### 2.10. Physico–Chemical Analysis

An infrared moisture analyzer (Model ID50, Marte Científica, Santa Rita do Sapucaí, Brazil) operating at 105 °C was used to measure the moisture content. Water activity (aw) was assessed at 25 °C using an AquaLab Decagon CX-2 m (Pullman, WA, USA) [35].

The method described by Manickavasagan et al. [36] was used to determine the powder’s solubility index. A volume of 100 mL of distilled water was mixed with approximately 1 g of the powder. A magnetic stirrer was used to agitate the mixture. The mixture was centrifuged (Nüve NF400R, Ankara City, Turkey) at 10,000 rpm for 15 min after being incubated for 30 min at 37 °C. A drying vessel was used to hold the precipitate, which was then dried at 105 °C until it was constantly weighed. The ratio of the solid mass of the supernatant to the initial sample weight (1 g) was used to calculate the solubility index (%).

At room temperature, a colorimeter (Minolta CM-3600j, Tokyo, Japan) was used to measure the powder’s color values. The CIELAB system scale was used to express the results, which comprise the brightness (L*), the green-to-red color scale (a*), and the blue-to-yellow color scale (b*).

### 2.11. Statistical Analysis

Triplicates of each experiment were implemented. Mean ± standard deviation (SD) was used to report the results. The two-way ANOVA was employed to analyze the influence of the plasma treatment variables (gas flow rate and exposure time) and enzymatic incubation (concentration and duration). One-way ANOVA was used to examine how drying conditions and storage stability affected each other. Significant differences between samples were identified using Duncan’s multiple range test (*p* < 0.05). SPSS for Windows, version 16 (SPSS Inc., Chicago, IL, USA) was employed to conduct all statistical analyses.

## 3. Results and Discussions

### 3.1. Cold Plasma Pretreatment for Enhancing the Extraction of Bioactive Ingredients

The results in Table 1 illustrate the correlation between the cold plasma pretreatment and the improvement in bioactive ingredient extraction from edamame. Following the CP treatment, the edamame extracts yielded genistein contents ranging from 3.28 to 3.69 mg/100 g, and TPCs and TFCs ranging from 5.88 to 15.0 mg GAE/100 g and 3.74 to 6.77 mg CAE/100 g, respectively. The optimal pretreatment conditions were found to be a gas flow rate of 5 L/min and a treatment duration of 30 min, resulting in the highest TPC (15.0 ± 0.37 mg GAE/100 g), TFC (6.77 ± 0.18 mg CAE/100 g), and genistein concentration of 3.69 ± 0.01 mg/100 g (Table 1). The plasma pretreatment promoted the release of free soluble phenolics from their bound forms by disrupting cell membranes, depolymerizing polymeric phenols, and generating oxidizing species. A comparable result was reported by Alu’datt et al. [37] who found that the concentrations of bound phenolics were higher than those of free phenolics in CP-treated soybeans, green soybeans, and soybean sprouts.

The bioactive compound content in the samples subjected to the optimal conditions was markedly greater (*p* < 0.05) compared to that from samples subjected to gas flow rates of 7 and 9 L/min with the same treatment duration. This finding is in line with research by Jan and Gavahian [38], who concluded that higher gas flow rates led to decreased anthocyanin contents and TPCs from blue pea flower extract. An excessive CP gas flow can minimize the extent of gas-mediated degradation for optimal extraction efficiency. Additionally, Paixao et al. [39] reported that the TPC decreased by 30% when the nitrogen gas flow rate was increased from 10 to 30 mL/min after 10 min of treatment. The plant cell degradation efficiency is decreased by a high gas flow rate, which dilutes the ozone effect caused by the ozone reaction in plasma. Because of the increased reactivity of oxygen in plasma, which encourages reactant interaction and speeds up the degradation reaction, the TPC, TFC, and genistein content degrade more readily at lower gas flow rates [38].

When comparing the 30-min to the 20-min plasma pretreatment, the bioactive compound content increased significantly (*p* < 0.05) only at a gas flow rate of 5 L/min. Many studies [40,41,42] have shown that extended treatment durations can more effectively disrupt plant materials, yielding more active compounds. Nevertheless, the extraction efficiency stops exhibiting a significant improvement if the treatment duration surpasses the optimal threshold at a suboptimal gas flow rate. This is due to the fact that cold plasma pretreatment has the potential to greatly damage the cell structure and improve the material’s surface hydrophilicity once the optimal treatment duration has been obtained [43]. Our findings further support the conclusion that plasma treatment effectively increased the concentrations of phytochemicals extracted from edamame.

### 3.2. Cold Plasma and Enzymatic Extraction for Bioactive Ingredients

In order to facilitate the release of intracellular compounds of interest, especially phenolic compounds, enzyme-assisted extraction exploits the ability of particular enzymes to catalyze the breakdown or modification of cellular walls. Two main factors that influence the bioactive compound content are the enzyme concentration and extraction time. In this study, pectinase, xylanase, and cellulase enzymes were used on edamame samples that were pretreated with the optimal CP treatment condition, identified as described in Section 3.1. The highest TPC, TFC, and genistein content were observed at a 2.0% (*v*/*v*) enzyme concentration with a 240-min incubation duration (Table 2).

The TPC and TFC increased when the enzyme PXC complex concentration was increased from 0% to 2.0% with a 240-min extraction duration. Ni-shad et al. [44] reported similar results, determining that the highest TPC levels were obtained after applying a 1% cellulolytic enzyme mixture concentration for 288.6 min. Table 2 also shows the effect of the enzyme concentration on the genistein content. The genistein content also increased at 1.0 and 1.5% enzyme levels, reaching 11.4 ± 0.35 and 12.5 ± 0.07 mg/100 g, respectively. However, at 2.0%, the genistein content plateaued. This may be due to an excessive enzyme concentration causing intense hydrolysis, releasing bioactive substances but then reaching a reaction equilibrium, where further increases in the enzyme do not enhance the yield [45]. According to Fu et al. [46], the yield of extraction is highest when every substrate molecule is attached to an active site in the enzyme. Beyond that, any excess enzyme does not improve outcomes and may even interfere with the process, making it inefficient and potentially inhibitory.

At a 2.0% (*v*/*v*) enzyme concentration, the extraction durations also affect the TPC, TFC, and genistein content (*p* < 0.05). Specifically, the total TPC, TFC, and genistein content increased to 22.5 ± 0.23 mg GAE/100 g, 15.3 ± 0.13 mg CAE/100 g, and 12.6 ± 0.10 mg/100 g, respectively, with a duration of 120 to 240 min. For the extraction process to yield high concentrations of genistein and other phenolics in the extracts, it is crucial to optimize the enzyme concentration and incubation time [47]. Generally, the incubation should be long enough to allow for greater degradation of cell wall components, which leads to higher bioactive compound release. These results indicate that longer incubation times significantly increased the extraction yield. Therefore, an incubation duration of 240 min with a 2.0% (*v*/*v*) enzyme concentration was chosen for the subsequent experiments.

### 3.3. Comparison of Spray-Drying and Freeze-Drying of Edamame Extract

The average yield, water activity, moisture content, TPC, TFC, genistein content, and antioxidant activities of the edamame extract powders generated through the spray-drying and freeze-drying methods are shown in Table 3. The biomass recovery was higher with spray-drying (68.0%) than with freeze-drying (43.4%). This increase can be attributed to improved heat and mass transfer during spray-drying. Sasikumar et al. [48] reported similar findings, stating that spray-drying produced the highest yield of blood fruit powder when compared to freeze- and tray-drying.

According to the productivity values, under the experimental conditions used in this study, spray-drying accomplished water removal in 2 h while freeze-drying took approximately 58 h to complete. This explains the higher productivity values associated with spray-drying [49]. Therefore, in this study, spray-drying demonstrated a superior speed, efficiency, and yield, making it more appropriate for encapsulating the crude isoflavone extract. In addition, Jovanović et al. [50] observed that freeze-drying is a time-intensive and expensive procedure, with a cost per kg of water evaporated that is approximately six times higher than that of spray-drying. Using hot air or inert gas, spray-drying can be used as an industrial procedure that turns a solution into powder.

The powder stability was contingent upon the moisture content and water activity of powder samples. The samples that were freeze-dried had a higher moisture content than those that were spray-dried following encapsulation. Freeze-drying (lyophilization) removes water by sublimation under low-pressure and -temperature conditions. While this method is gentle and preserves heat-sensitive compounds, it is generally less efficient in removing moisture. The residual moisture content in freeze-dried products can be higher due to the incomplete removal of bound water during the secondary drying phase [51]. Furthermore, the sublimation of ice crystals frequently results in the formation of a porous structure in freeze-dried powders. This porosity can lead to higher moisture retention as water vapor may be trapped within the matrix [52]. The strong contact between the particles and the hot air may have contributed to the spray-dried method’s superior water removal capabilities. Using maltodextrin as a carrier, Valková et al. [53] also noticed a similar finding with spray-dried β-glucan powder (*Pleurotus ostreatus*). The powder’s water activity and moisture content appeared to be reduced more effectively by the spray-drying method, suggesting that this powder was more stable than the one produced with the freeze-drying method.

Similarly to industrial drying products, the water activity values of the powder samples produced through both drying methods varied between 0.36 and 0.37. In general, the water activity values of all of the samples were within what was anticipated for powdered products and within the suggested thresholds for guaranteeing microbiological stability (<0.6) [54].

Table 3 displays the bioactive compound data pertaining to the drying duration of the extracted powder using two distinct drying techniques. Compared to the freeze-dried powder, the spray-dried powder had a significantly (*p* < 0.05) higher TPC and TFC. Since the maximum time taken to dry the extracts using the freeze-dryer was 58 h, this can lead to TPC and TFC losses due to prolonged exposure, even with the low temperatures and vacuum conditions within the freeze-dryer, causing potential degradation over time [55]. Another possible reason could be the addition of maltodextrin to the extract solution, as maltodextrin has been reported to contribute to the preservation and stabilization of the TPC and TFC during the drying process. Sasikumar et al. [48] reported that maltodextrin, acting as a drying agent, forms an active layer that shields polyphenols from heat and oxidation. Freeze-drying could preserve genistein, which is associated with the TEAC, by inhibiting biological activity. However, processing at a low temperature did not significantly affect the genistein content in the extract powder compared to that in the spray-dried powder.

### 3.4. Morphological Observations

**Figure 1:**
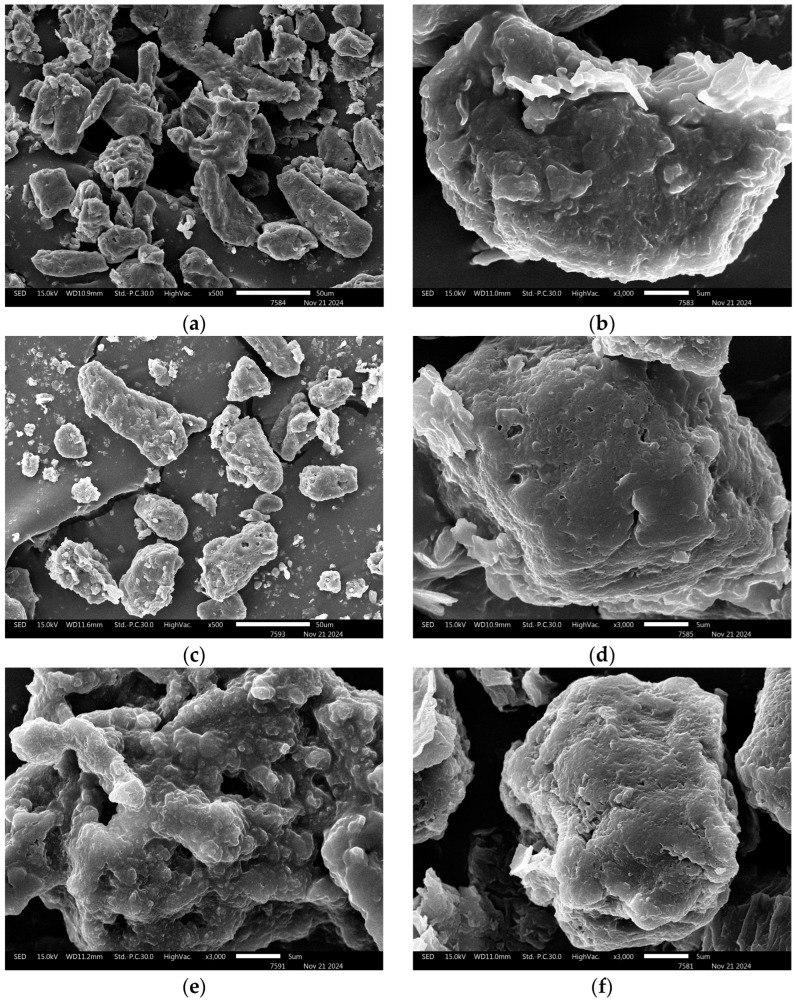
Scanning electron microscope (SEM) images of (**a**,**b**) untreated, (**c**,**d**) cold plasma-pretreated, and (**e**,**f**) cold plasma- and enzyme-treated dried edamame extracts. The 500× magnification SEM images (15 kV, 11.6 mm) are displayed in the left column. The 3000× magnification SEM images (15 kV, 11.0 mm) are displayed in the right column.

SEM was used to observe the dried extracts that were untreated, CP-pretreated, and combination-treated. The micrographs of the untreated samples exhibited smooth surfaces with intact structures, as shown by the SEM images in Figure 1. The surface of the powder particles treated with CP, however, was coarse, and the combined treatment led to the formation of numerous hollow pores. As seen in Figure 1c, the CP–enzyme combined treatment caused significant structural changes, indicated by the deeper pores formed on the powder granules’ surfaces. These findings are consistent with earlier research that found no intact cell wall structure in cornstarch subjected to a combination of cold plasma and enzymatic hydrolysis. This was attributed to direct damage to the epidermal tissue cell wall caused by plasma [56]. Additionally, the plasma treatment altered the plant tissues’ surface morphology, inducing cracks in the cornstarch and resulting in a porous structure with higher absorption capacity [56]. It was also observed by Huang et al. [57] that plasma treatments increased the hydrophilicity of the surfaces of fresh lettuce and white grapes by degrading the cuticle layer, which comprises a mixture of hydrophobic waxes.

In order to facilitate phenolic compound recovery from biomass, CP causes surface modifications and structural damage that decrease the internal molecular diffusion resistance and improve the extractability of hydrophilic compounds [58]. A cold plasma pretreatment method was developed in the present research to improve TPC, TFC, and genistein extraction from edamame powder, based on CP’s demonstrated capacity to modify biomass by disrupting cell walls and increasing the surface hydrophilicity. A correlation between the cold plasma-induced microstructural changes (Figure 1b,c) and the increased bioactive compound extraction efficiency (Table 1) was clearly demonstrated.

### 3.5. Storage Stability of Spray-Dried Extract Powder

The results for the storage stability of the physical properties and phytochemical compounds in the crude extract powder at different storage temperatures and durations are shown in Table 4. Water solubility is one of the most utilized parameters to verify the capacity of a powder to maintain a homogenous mixture with water [59]. The value of the water solubility was significantly reduced compared to that of the control (Day 0), ranging from 83.15% to 84.80% at both temperature levels during storage. The decrease in the powder sample solubility over time in storage might be attributed to the formation of a complex, cross-linked maltodextrin matrix, which can restrict water transport and powder particle hydration, leading to changes in the powder’s characteristics and potentially impacting its functionality [60]. The behavior of edible powders when reconstituted in water is significantly influenced by their solubility, a critical functional characteristic. The extract powder prepared with only 20% maltodextrin in this study could maintain a high solubility (more than 80%) over an extended storage duration. One of the most common ingredients used as a wall material for spray-drying plant extracts is maltodextrin because of its high water solubility [61].

Initially, the extract powder’s color parameters (L*, a*, and b*) were 90.63, −0.69, and 4.39 for storage at 4 °C, and 91.01, −0.83, and 4.82 for storage at 25 °C, respectively. Storage at 4 and 25 °C for 45 days led to a slight increase in lightness (L*) to 92.44 and 92.90, accompanied by a decrease in b* values across all of the tested powders. The color change was significantly (*p* < 0.05) influenced by the storage period, whereas the storage temperature had a minimal impact. The corresponding rise in a* values confirms the visually noticeable green coloration increase in all of the dried powders at different storage temperatures. However, the breakdown of natural pigments, including carotenoids and chlorophyll derivatives, which provide edamame extract powder its yellow color, is the reason for the observed decrease in yellowness (b* value) after 45 days of storage at both 4 °C and 25 °C [62]. Moreover, Maillard reactions and other non-enzymatic browning processes can occur during storage, leading to the formation of brown pigments that overshadow the yellow hues, thereby decreasing the b* value [63].

Variations in the TPC and TFC were observed throughout the 45-day storage period, which were influenced by the storage temperature. The best stability, with over 95% retention, was demonstrated by the TPC and TFC of the dried extract kept at 4 °C without exposure to light (Table 4). However, storage at 25 °C retained 86% of the TPC and 58% of the TFC. A notable reduction in the TPC and TFC was seen with increased storage time. In the vacuum packages, the oxygen content progressively rises from 0% to 10%, according to Moradinezhad and Dorostkar [64]. Therefore, the decline in the total phenolics during storage may be due to the oxidation of polyphenols.

The effects of time and temperature were examined during 45 days of storage by measuring the antioxidant activity, genistein levels, and quantitative flavonoid content every 15 days. The antioxidant activity and flavonoid content were lower at 25 °C than at 4 °C, the lower storage temperature. An elevated temperature could cause the degradation of some types of flavonoids with antioxidant activity reduction. According to a review by Rahayu et al. [65], the storage temperature has an impact on antioxidant capacity and flavonoid concentrations. Elevated storage temperatures can induce various mechanisms that lead to flavonoid decomposition. During the 30-day storage period, the maximum flavonoid content and antioxidant activity recorded at the initial time point progressively declined under the same storage temperature conditions. Nevertheless, over 60% of the bioactive compounds and antioxidant activity was preserved.

During storage, the genistein content exhibited minimal variations under most conditions at both 4 and 25 °C, with values spanning from 5.54 to 6.77 mg/100 g. The only condition that did not significantly affect the genistein content was storage at 4 °C, which maintained over 95% stability of the genistein throughout the storage period. In contrast, storage at 25 °C resulted in a substantial reduction in the genistein content from the initial value to 5.54 ± 0.04 mg/100 g after 45 days. However, no significant reduction was observed between days 15 and 45 of storage at 25 °C. The conversion of malonyl to acetyl isoflavones, which were not measured in this study, may account for a loss of this magnitude because isoflavones are broken down into other forms under stressors. The genistein and total flavonoid levels decreased the antioxidant activity, and these levels varied significantly (*p* < 0.05) over the course of storage. Similarly, studies have found that higher storage temperatures and prolonged storage durations led to a decline in the bioactive content and antioxidant activity of isoflavone crude extract powder.

The results of this study demonstrate that storing the crude extract powder at 4 °C can significantly stabilize its TPC, TFC, genistein content, and antioxidant activity while preserving its physical characteristics (color and solubility) and phytochemicals over time.

## 4. Conclusions

The current study shows that the recovery of bioactive compounds from edamame is greatly improved when CP pretreatment and enzymatic extraction are combined. The structural modifications induced by this combined method improved cell wall disruption, contributing to enhanced extractability. Among the drying techniques evaluated, spray-drying proved to be more effective in preserving key phytochemicals and ensuring product stability during storage. Overall, this synergistic extraction–drying approach offers a viable strategy for developing high-quality, isoflavone-rich powders suitable for use in functional food applications.

## Figures and Tables

**Table 1:** Effects of gas flow rate and time of cold plasma pretreatment on bioactive compounds in edamame.

Cold Plasma Pretreatment		Bioactive Compounds		
Gas Flow Rate (L/min)	Time (min)	TPC (mg GAE/100 g)	TFC (mg CAE/100 g)	Genistein (mg/100 g)
Control	0	8.75 ± 0.76 ^d^	1.64 ± 0.10 ^f^	1.46 ± 0.14 ^g^
5 L/min	20	5.88 ± 0.43 ^e^	4.04 ± 0.19 ^d^	3.28 ± 0.09 ^f^
	30	15.0 ± 0.37 ^a^	6.77 ± 0.18 ^a^	3.69 ± 0.01 ^a^
7 L/min	20	14.1 ± 0.65 ^ab^	4.00 ± 0.24 ^d^	3.49 ± 0.01 ^d^
	30	13.2 ± 0.61 ^b^	4.39 ± 0.15 ^c^	3.45 ±0.08 ^e^
9 L/min	20	12.2 ± 0.35 ^c^	3.74 ± 0.07 ^e^	3.52 ± 0.01 ^c^
	30	8.17 ± 0.54 ^d^	5.27 ± 0.32 ^b^	3.59 ± 0.01 ^b^

Means ± standard deviation (n = 3) are used to express the data. Statistically significant differences (*p* < 0.05) are indicated by different letters (a–g) in the same column.

**Table 2:** Effect of cold plasma and enzymatic extraction on bioactive compounds in edamame.

Enzyme Treatment		Bioactive Compounds		
Concentration (%)	Time (min)	TPC (mg GAE/100 g)	TFC (mg CAE/100 g)	Genistein (mg/100 g)
Control	0	13.0 ± 0.48 ^f^	3.45 ± 0.01 ^f^	3.09 ± 0.09 ^d^
1.0	120	20.4 ± 0.89 ^bc^	14.2 ± 0.64 ^c^	10.7 ± 0.28 ^c^
	180	20.9 ± 0.88 ^bc^	15.1 ± 0.39 ^b^	11.3 ± 0.39 ^b^
	240	16.6 ± 0.31 ^d^	12.8 ± 0.90 ^de^	11.4 ± 0.35 ^b^
1.5	120	20.0 ± 0.43 ^bc^	11.9 ± 0.84 ^e^	10.9 ± 0.31 ^c^
	180	14.6 ± 0.31 ^e^	12.8 ± 0.28 ^de^	10.6 ± 0.44 ^c^
	240	16.8 ± 0.97 ^d^	13.1 ± 0.39 ^d^	12.5 ± 0.07 ^a^
2.0	120	19.5 ± 1.06 ^c^	11.0 ± 0.42 ^e^	10.7 ± 0.39 ^c^
	180	21.1 ± 0.58 ^b^	15.0 ± 0.18 ^b^	11.7 ± 0.31 ^b^
	240	22.5 ± 0.23 ^a^	15.3 ± 0.13 ^a^	12.6 ± 0.10 ^a^

Means ± standard deviation (n = 3) are used to express the data. Statistically significant differences (*p* < 0.05) are indicated by different letters (a–f) in the same column.

**Table 3:** Effect of different drying methods on bioactive compounds extracted from edamame.

Drying Methods	Powder Yield (%)	Moisture Content %	Water Activity	TPC (mg GAE/100 g)	TFC (mg CAE/100 g)	Genistein (mg/100 g DM)	Antioxidant Activities	
							FRAP (mg Fe (II)/g)	TEAC (mg Trolox/g)
Freeze-drying	43.4 ± 0.15 ^b^	7.82 ± 0.19 ^a^	0.36 ± 0.00 ^a^	23.6 ± 0.45 ^b^	6.99 ± 0.09 ^b^	5.57 ± 0.02 ^a^	13.0 ± 0.14 ^b^	56.7 ± 1.57 ^a^
Spray-drying	68.0 ± 0.19 ^a^	5.74 ± 0.23 ^b^	0.37 ± 0.00 ^a^	35.9 ± 0.79 ^a^	9.53 ± 0.09 ^a^	5.19 ± 0.01 ^a^	18.2 ± 0.18 ^a^	43.9 ± 2.10 ^b^

Means ± standard deviation (n = 3) are used to express the data. Statistically significant differences (*p* < 0.05) are indicated by different letters (a–b) in the same column.

**Table 4:** Effect of storage temperature and time on the physical properties, bioactive compound, and antioxidant activities of spray-dried edamame extract powder.

Storage Temperature (°C)	Time (Days)	Physical Properties				TPC (mg GAE/100 g)	TFC (mg CAE/100 g)	Genistein (mg/100 g)	Antioxidant Activities	
		Solubility (%)	Color							
			L*	a*	b*				FRAP (mg Fe (II)/g)	TEAC (mg Trolox/g)
4	0	84.70 ± 1.62 ^a^	90.63 ± 0.11 ^b^	−0.79 ± 0.05 ^a^	4.39 ± 0.15 ^a^	32.22 ± 0.27 ^a^	13.36 ± 0.10 ^a^	6.77 ± 0.32 ^a^	49.66 ± 0.11 ^a^	95.44 ± 1.93 ^a^
	15	83.05 ± 1.67 ^b^	90.55 ± 0.12 ^b^	−0.77 ± 0.03 ^a^	4.28 ± 0.30 ^a^	31.20 ± 0.43 ^ab^	13.33 ± 0.20 ^a^	6.73 ± 0.38 ^a^	48.39 ± 1.80 ^a^	91.49 ± 1.33 ^a^
	30	83.82 ± 1.80 ^b^	91.96 ± 0.19 ^a^	−0.76 ± 0.03 ^a^	4.27 ± 2.52 ^a^	29.73 ± 1.01 ^c^	8.78 ± 0.05 ^b^	6.55 ± 0.36 ^a^	49.09 ± 0.29 ^a^	89.34 ± 1.01 ^b^
	45	83.74 ± 1.27 ^b^	92.44 ± 0.46 ^a^	−0.74 ± 0.04 ^a^	4.18 ± 0.48 ^b^	29.14 ± 0.42 ^c^	6.82 ± 0.19 ^c^	6.43 ± 0.41 ^a^	46.89 ± 0.65 ^b^	84.70 ± 1.42 ^b^
25	0	84.80 ± 2.55 ^a^	91.01 ± 0.13 ^a^	−0.83 ± 0.06 ^b^	4.52 ± 0.49 ^a^	32.12 ± 0.22 ^a^	13.26 ± 0.03 ^a^	6.70 ± 0.22 ^a^	48.14 ± 0.29 ^a^	92.08 ± 1.39 ^a^
	15	83.72 ± 0.54 ^b^	92.26 ± 0.94 ^a^	−0.78 ± 0.04 ^a^	4.47 ± 0.07 ^a^	26.58 ± 0.27 ^d^	8.01 ± 0.11 ^b^	5.73 ± 0.79 ^b^	43.93 ± 0.11 ^c^	82.21 ± 0.59 ^b^
	30	83.15 ± 1.20 ^b^	92.50 ± 0.39 ^a^	−0.75 ± 0.02 ^a^	4.44 ± 0.12 ^a^	24.78 ± 0.70 ^e^	8.40 ± 0.58 ^b^	5.60 ± 0.48 ^b^	42.14 ± 0.19 ^c^	66.82 ± 1.30 ^d^
	45	83.37 ± 0.33 ^b^	92.90 ± 0.28 ^a^	−0.77 ± 0.08 ^a^	4.16 ± 0.25 ^b^	20.34 ± 0.34 ^f^	6.64 ± 0.05 ^c^	5.54 ± 0.04 ^b^	36.53 ± 0.11 ^d^	55.48 ± 1.74 ^e^

Means ± standard deviation (n = 3) are used to express the data. Statistically significant differences (*p* < 0.05) are indicated by different letters (a–e) in the same column.

## Data Availability

The data presented in this study is available on request from the corresponding author.

## Abbreviations

CAE	catechin equivalents
CP	cold plasma
DPPH	2,2-diphenyl-1-picryl-hydrazyl
FRAP	ferric-reducing antioxidant power
GAE	gallic acid equivalents
HPLC	high-performance liquid chromatography
SEM	scanning electron microscopy
TEAC	trolox equivalent antioxidant capacity
TFC	total flavonoid content
TPC	total phenolic content
TPTZ	2,4,6-Tris (2-pyridyl)-1,3,5-triazine
UV	ultraviolet radiation
